# LINC00883 Promotes Drug Resistance of Glioma Through a microRNA-136/NEK1-Dependent Mechanism

**DOI:** 10.3389/fonc.2021.692265

**Published:** 2022-01-10

**Authors:** Yongzhe Li, Xin Gao

**Affiliations:** ^1^ Department of Neurosurgery, The Second Affiliated Hospital of Harbin Medical University, Harbin, China; ^2^ Department of Neurosurgery, The Affiliated Hospital of Qingdao University, Qingdao, China

**Keywords:** long noncoding RNA, LINC00883, microRNA-136, NIMA-related kinase 1, glioma, multidrug resistance

## Abstract

**Objective:**

Accumulating evidence has highlighted the roles of long noncoding RNAs (lncRNAs) as competing endogenous RNAs (ceRNAs) of microRNAs (miRNAs) through their binding sites in the progression of glioma. Hereby, we aim to explore the role of LINC00883 as a regulator of miR-136 and its target, NIMA-related kinase 1 (NEK1), thus, its involvement in the drug resistance of glioma cells.

**Methods and Results:**

Mechanistic investigations by dual-luciferase reporter, RNA pull-down, and RNA-binding protein immunoprecipitation (RIP) assays indicated that LINC00883 bound to miR-136, thereby blocking miR-136-induced downregulation of NEK1. Through gain-of-function experiments in U251 cells that presented a high drug resistance, we found that ectopic expression of LINC00883 resulted in increased MRP (encoding multidrug resistance-associated protein), limited cell apoptosis, and increased proliferation. Expectedly, depleting LINC00883 yielded tumor-suppressive and anti-chemoresistance effects on U251 cells by increasing miR-136 and inhibiting NEK1. Next, drug-resistant glioma cell line SOWZ1, drug-sensitive glioma cell line SOWZ2, and drug-resistant glioma cell line SOWZ2-BCNU (SOWZ2 cultured in BCNU) were applied to validate the roles of LINC00883 in the regulation of multidrug resistance. LINC00883 knockdown suppressed the viability of SWOZ1, SWOZ2, and SWOZ2-BCNU cells.

**Conclusion:**

In conclusion, LINC00883 knockdown reduces drug resistance in glioma. Hence, our study provides a future strategy to prevent drug resistance-induced therapeutic failure in glioma.

## Introduction

Gliomas are heterogeneous tumors that originate from the glial cells and have been regarded as the most fatal form of brain cancer (1). Based on clinical statistics, approximately 50% of all glioma cases are malignant in nature (2), and 80% of brain tumors diagnosed are gliomas in adults, with no discrepancies of gender or ethnicity (3). According to World Health Organization 2016 classification, there are several different histological subtypes of glioma such as astrocytomas, oligodendrogliomas, and glioblastomas that refer to grade IV glioma (4). The invasiveness of glioma is marked by its potential to inflict incurable pain on patients (5). Glioma manifests itself as a primary tumor and has a high mortality due to the inefficacy of current treatment protocols, which include chemotherapy against which gliomas developed resistance (6). More recently, accumulating evidence has been presented by various investigations conducted into novel, more effective glioma therapy methods (3, 7). A recent study has revealed that potential treatment regimens could be developed through the connection between the regulators of the cellular process (growth, differentiation, proliferation, and apoptosis) (5). Thus, it is of great significance that investigations are opened into such markers that are implicated in key tumorigenic events in order to identify more effective glioma treatment methods.

Long noncoding RNAs (lncRNAs) are transcripts longer than 200 nucleotides that are involved in a variety of biological processes. Reports have indicated that the alteration in the expression of lncRNAs may be essential in the incidence and progression of various diseases (8, 9), in particular, with LINC00883 reportedly playing a role by binding to microRNA-150 (miR-150) (10). Some lncRNAs have been identified as competing endogenous RNAs (ceRNAs) for specific miRNAs (11). miRNAs are noncoding RNA molecules that function on a posttranscriptional level by regulating gene expression and an array of other cellular process and also act as oncogenes or tumor suppressors in certain cancers, including glioma (12). Researchers have proposed that a variety of miRNAs are associated with glioma, with existing evidence showing that downregulated miR-34a expression might result in poor prognosis. Some reports have intriguingly suggested a negative correlation between miR-136 and the survival and malignancy of glioma (12–14). However, at present, a scarcity of literature exists pertaining to whether miR-136 is involved in the development of glioma in combination with LINC00883, which was predicted to bind to miR-136 by the RNA22 website. In addition, mitosis A (NIMA)-related kinase 1 (NEK1), belonging to NEK family, has never yet been reported to be involved in the modulation of mitosis and apoptosis, processes of which are involved in the regulation of tumor progression (15). Moreover, the facilitating effects of the high expression of NEK1 in regard to resistance to chemotherapy in the treatment of renal cell carcinoma have been demonstrated in a previous study (16). Based on the aforementioned findings, we hypothesized that LINC00883 might bind to miR-136 with involvement of NEK1 in glioma cells, by which tumor progression is regulated. To test this hypothesis, we analyzed the effects of LINC00883/miR-136/NEK1 on glioma cell resistance to temozolomide (TMZ) and tumorigenicity in the *in vitro* and *in vivo* settings.

## Materials and Methods

### Ethics Statement

The protocols of the present study were all approved by the Ethics Committee of the Second Affiliated Hospital of Harbin Medical University. Written informed consents were signed and obtained from all patients or their families. The study was conducted with the approval of the Animal Ethics Committee of the Second Affiliated Hospital of Harbin Medical University.

### *In Silico* Analysis

With “glioma” used as the key word, microarray data related to glioma (GSE15824 and GSE4290) and their annotated probe files were downloaded from the Gene Expression Omnibus (GEO) database (https://www.ncbi.nlm.nih.gov/geo/), and the microarray data were obtained by Human Genome U133 Plus 2.0 Array (Affymetrix, Inc., CA, USA). GSE15824 microarray data included two normal tissues and 12 glioma tissues, while GSE4290 microarray data included 23 normal tissues and 157 glioma tissues. The Affy package of R software was applied for background correction and normalization of each microarray data (17). Next, the linear models and empirical Bayes method combined with traditional *t*-test of Limma package were employed for nonspecific filtration for the screening of the differentially expressed genes (DEGs) and lncRNAs (18). DEGs in glioma of The Cancer Genome Atlas (TCGA) database were screened using the Gene Expression Profiling Interactive Analysis 2.0 (GEPIA2) website (http://gepia2.cancer-pku.cn/#index) and then intersected with the predicted results obtained from the GSE15824 and GSE4290 microarray data using jvenn website (http://jvenn.toulouse.inra.fr/app/example.html?tdsourcetag=s_pcqq_aiomsg) to determine the studied lncRNA. The GEPIA2 website was applied to identify the mRNA co-expressed with the target lncRNA. Finally, the RNA22 website (https://cm.jefferson.edu/rna22/) was used to predict the miRNAs that bound to lncRNAs and mRNAs.

### Clinical Sample Collection

Tumor tissues were harvested from 31 glioma patients (19 males and 12 females with a mean age of 53.4 ± 1.2 years) undergoing surgical resection from October 2017 to October 2018 at the Second Affiliated Hospital of Harbin Medical University. None of the enrolled patients had received radiotherapy or chemotherapy prior to the experiment, and they did not suffer from dysfunction of organs such as heart, lung, liver, and kidney. Patients with distant metastasis or other malignant tumors were excluded from the study. Meanwhile, 12 specimens were collected from brain trauma patients and pathologically confirmed to be non-neoplastic brain tissues.

### Cell Culture

Five human glioma cell lines {A-172, CHG-5 [both from American Type Culture Collection (ATCC), Manassas, VA, USA], U251, T98G, and SHG-44 [these three cell lines from Procell, Wuhan, China]} and human embryonic kidney cells 293T (ATCC) were cultured in Dulbecco’s modified Eagle’s medium encompassing 10% fetal bovine serum (FBS) in a 5% CO_2_ incubator at 37°C. Logarithmically growing U251 cell line was seeded into a 96-well plate at a density of 1 × 10^5^ cells/ml in each well. After 24-h culture, the cells received treatment with 50 μM TMZ for 1, 24, 36, 72, and 96 h, respectively. Afterward, the expression of multidrug resistance-associated protein (MRP), LINC00883, and miR-136 was determined in each cell line accordingly.

### Lentiviral Vector Construction and Cell Treatment

Based on the known sequences of LINC00883 and miR-136 in National Center for Biotechnology Information (NCBI), Hanbio Biotechnology Co., Ltd. (Shanghai, China), was commissioned to construct overexpression or silencing lentiviral vectors.

To alter LINC00883 expression, the cells were not transduced as control cells, and other cells were transduced with overexpression-negative control (oe-NC), oe-LINC00883, short hairpin RNA (sh)-NC, or sh-LINC00883. To alter miR-136 expression, the cells were not transduced as control cells, and other cells were transduced with NC mimic, miR-136 mimic, NC inhibitor, and miR-136 inhibitor.

Cells at passage 3 were prepared into cell suspension, 2 × 10^5^ cells/ml of which were completely mixed with polybrene (6 μg/ml) and virus and then incubated at 37°C or centrifuged at 150 g and room temperature for 4 h. After 4 h, an equal volume of fresh medium was added to dilute the polybrene, and the cells were cultured for 3–4 days. Next, the medium was renewed, and the cells were passaged depending on cell growth for the subsequent experiments.

### Reverse Transcription Quantitative Polymerase Chain Reaction

The total RNA was extracted according to the instructions provided on the miRNeasy Mini Kit (217004, QIAGEN, Germany). The primers were designed and synthesized by Takara Biotechnology Ltd. (Dalian, Liaoning, China), as illustrated in **Supplementary Table S1**. RNA was then reversely transcribed into cDNA using the PrimeScript RT Kit (RR036A, Takara Biotechnology Ltd., Dalian, Liaoning, China). The cDNA was collected for fluorescent quantitative PCR in accordance with the instructions of the SYBR TaqTM II Kit (RR820A, TaKaRa Biotechnology Ltd., Dalian, Liaoning, China). The ABI7500 quantitative PCR instrument (7500, ABI Company, Oyster Bay, NY, USA) was introduced for real-time fluorescence quantitative PCR detection purposes. The relative expression was calculated using the 2^-△CT^ method with glyceraldehyde-3-phosphate dehydrogenase (GAPDH) or U6 used as the internal reference.

### Western Blot Analysis

Cells in the logarithmic growth phase were centrifuged for 20 min with the supernatant discarded and lysed with 100 μl lysis buffer and 1 μl proteinase inhibitors on ice for 30 min, followed by centrifugation at a low temperature for 10 min. Next, 50 μg protein was dissolved in 2× sodium dodecyl sulfate (SDS) loading buffer, boiled at 100°C for 5 min, and transferred to the polyvinylidene fluoride (PVDF) membrane after 10% SDS-polyacrylamide gel electrophoresis (SDS-PAGE). The PVDF membrane was blocked with 5% skimmed milk powder at room temperature for 1 h. After 2 min of PBS washing, the PVDF membrane was incubated with diluted antibody primary mouse polyclonal antibodies (Abcam, Cambridge, UK) against NEK1 (1:1,000, ab229489), proliferating cell nuclear antigen (PCNA; 1 g/ml, ab29), MRP (1:20, ab24102), B-cell lymphoma-2 (Bcl-2; 1:500, ab692), and Bcl-2-associated protein X (Bax; 1:1,000, ab32503) at 4°C overnight. The following day, the membrane was incubated with secondary antibody horseradish peroxidase (HRP)-labeled goat anti-mouse immunoglobulin G (IgG; 1:100, HA1003, Yanhui Biotechnology Co., Ltd., Shanghai, China) for 1 h. The membrane was then immersed in the electroluminescence (ECL) solution (ECL808-25, Biomiga, USA) at room temperature for 1 min. The ratio of the gray value of the target band to GAPDH was representative of the relative protein expression. The experiment was conducted in triplicate.

### Immunofluorescence

After 24 h of transfection, the cells were fixed in 4% paraformaldehyde for 15~20 min at room temperature, washed twice with PBS, and blocked in 2% calf serum for 20 min, after which the sealant was removed. Next, the cells were added with 2.5 ml green fluorescent-labeled NEK1 antibody (1:100, ab229489, Abcam) and cooled at 4°C for 30 min with the avoidance of light. After being washed twice with PBS, the cells were mounted with glycerol. The results were observed under a fluorescence microscope. The experiment was conducted in triplicate.

### RNA Fluorescence *In Situ* Hybridization

The cellular sublocalization of LINC00883 was determined using the Fluorescence *In Situ* Hybridization (FISH) Kit (C10910, Guangzhou RiboBio Co., Ltd., Guangzhou, China). The coverslips were placed on the bottom of the 24-well plate, and the cells in the logarithmic phase were detached from the coverslips (about 6 × 10^4^ cells/well). Once cell confluence reached 60%~70%, the cells were fixed in 4% paraformaldehyde for 10 min at room temperature and permeabilized with 1 ml of pre-cooled permeable fluid at 4°C for 5 min. Following the removal of the permeabilization fluid, the cells in each well were added with 200 μl of prehybridization solution and blocked at 37°C for 30 min. During prehybridization, the prehybridization solution was preheated at 37°C. Next, 2.5 μl FISH Probe Mix stored solution was added to the hybridization solution, avoiding exposure to light. Once the prehybridization solution was discarded in each well, the cells were hybridized with hybridization solution containing probes overnight at 37°C, avoiding exposure to light. The cells were then stained with 4’,6-diamidino-2-phenylindole (DAPI) for 10 min and mounted with a medium for fluorescence detection. The LINC00883-specific probe (**Supplementary Table S2**) was prepared by RiboBio (Guangzhou, Guangdong, China).

### Dual-Luciferase Reporter Assay

The binding sites between LINC00883 and miR-136 and between NEK1 and miR-136 were predicted using the biological prediction website microRNA.org. PmirGLO Vector (Promega Corporation, Madison, WI, USA) was used to construct the wild-type (WT) pmirGLO-LINC00883 vector and mutant (MUT) pmirGLO-LINC00883 vector. A full-length LINC00883 was inserted between the two enzyme sites, Xho I and Xba I. Following amplification, the products were transformed into the *Escherichia coli* DH5α cells. On the basis of pmirGLO-LINC00883-WT vector, the MUT vector containing mutated miR-136 binding site was constructed, with the recombinant plasmids transformed and amplified. According to the binding sequence between NEK1 mRNA 3′-untranslated region (UTR) and miR-136, the WT and MUT sequences were designed. The target sequence was chemically synthesized with the restriction sites Xho I and Not I, which were added onto both ends of the sequence, respectively. The synthesized fragment was cloned into the PUC57 vector. Once the positive clones were identified, the recombinant plasmids were identified using the DNA sequencing method. The plasmid was then subcloned into the psiCHECK-2 vector and transformed into *Escherichia coli* DH5α cells for amplification. The plasmid was transfected into 293T cells for 48 h. Then, cells were centrifuged at 12,000 rpm for 1 min, followed by supernatant collection. The effects of miR-136 on luciferase activity of LINC00883 or NEK1 3′-UTR were detected in accordance with the instructions of dual-luciferase activity detection kit from GeneCopoeia Inc. (D0010, purchased from Beijing Solabio Life Sciences Co., Ltd., Beijing, China). Glomax20/20 luminometer from Promega (E5311, purchased from Shaanxi Zhongmei Biotechnology Co., Ltd., Shaanxi, China) was utilized for luminance determination. The experiment was repeated three times.

### RNA Pull-Down and RNA-Binding Protein Immunoprecipitation

The cells were transfected with 50 nM biotin-labeled WT-bio-miR-136 and MUT-bio-miR-136 for 48 h. The cells were then collected, washed with PBS, and incubated in the specific lysis buffer (Ambion, Austin, TX, USA) for 10 min. The lysates were incubated with the M-280 streptavidin beads (S3762, Sigma-Aldrich, St. Louis, MO, USA) that were precoated with RNase-free bovine serum albumin (BSA) and yeast tRNA (TRNABAK-RO, Sigma, USA). After being incubated at 4°C for 3 h, the beads were washed twice with precooled lysis buffer, three times with low-salt buffer, and once with high-salt buffer. The combined RNAs were purified, and the enrichment of LINC00883 was detected by qPCR.

The cells were treated with lysis buffer (25 mM Tris-HCl pH = 7.4, 150 mM NaCl, 0.5% NP-40, 2 mM EDTA, 1 mM NaF, and 0.5 mM dithiothreitol) containing RNasin (Takara Biotechnology Ltd., Dalian, Liaoning, China) and protease inhibitors (B14001a, Roche, USA). The lysate was centrifuged at 12,000 g for 30 min, after which the supernatant was collected. The beads conjugated with antibody against Ago-2 (BMFA-1, Biomarker Technologies, Beijing, China) were subsequently added into lysate, while the beads conjugated with IgG were added in the negative control group. After 4 h of incubation at 4°C, the beads were washed three times with washing buffers (50 mM Tris-HCl, 300 mM NaCl pH = 7.4, 1 mM MgCl 2, and 0.1% NP-40). RNA was extracted from magnetic beads, and LINC00883 expression was detected by qPCR.

### Colony Formation Assay

Cells in the logarithmic growth phase were inoculated in 75-mm plate (single cell ratio >95% in cell suspension), with 800 cells per plate. After 24 h of culture, the cells were placed in the plates in a respective manner, followed by an additional 9 h of culturing. Once the culture liquid was removed, the cells were washed twice with phosphate buffer (pH = 6.8), fixed in methanol for 20 min, and stained with 10% Giemsa for 20 min, followed by washing with deionized water and subsequent drying. The number of colonies was calculated under an anatomic microscope with 20 or more cells per plate. The colony formation rate was calculated, and the experiment was conducted in triplicate.

### 3-(4,5-Dimethylthiazol-2-yl)-2,5-Diphenyltetrazolium Bromide Assay

Cells from the culture bottles were inoculated into 96-well plates and cultured in a complete medium composed of 1.0 mM adenosine triphosphate (ATP) for 12, 24, 48, and 72 h, respectively. The growth vitality of the 3T3-LI preadipocytes was measured at each time point. At the corresponding time points, 10 µl of 3-(4,5-dimethylthiazol-2-yl)-2,5-diphenyltetrazolium bromide (MTT) solution was added to each well, and the plates were placed back into the cell incubator for another 4 h of culture. At the end of the culture process, the plates with MTT were removed and followed by the addition of 150 µl dimethyl sulfoxide (DMSO) to each well and cell culture. The culture process was terminated when the blue crystal in each well was dissolved in DMSO. The optical density (OD) values were measured at a wavelength of 570 nm using a microplate reader.

### 5-Ethynyl-2′-Deoxyuridine Cell Proliferation Assay

Here, 5-ethynyl-2′-deoxyuridine (EdU) solution was added to the culture plates, which were then incubated at room temperature for 2 h and washed with PBS. After being fixed with 40 g/L paraformaldehyde for 30 min, the cells were incubated for 8 min in the glycine solution, washed by PBS, and then rinsed with PBS containing Triton X-100 with a volume fraction of 0.5%. Then, the cells were incubated with Apollo^®^ staining solution at room temperature for 30 min under conditions void of light. After being washed with methanol and PBS, respectively (two times each), the cells were added with Hoechst 3334 reaction liquid (1×), incubated at room temperature for 20 min with the avoidance of light, and observed under a fluorescence microscope. Subsequently, three fields of view were selected from the visual field (×400), with the number of EdU-stained cells (proliferative cells) and Hoechst 33342-stained cells (total cells) calculated. Cell proliferation rate was then calculated using the following formula: Cell proliferation rate = the number of proliferative cells/the number of total cells × 100%. The experiment was conducted in triplicate.

### Flow Cytometry

Propidium iodide (PI) single staining method was used to detect cell cycle. After 48 h of transfection, the cells were collected, treated with 0.25% trypsin at 4°C, and centrifuged for 5 min, after which the supernatant was discarded. After being washed three times with cold PBS, the cells were centrifuged again, with the supernatant removed accordingly. PBS was used for cell resuspension, and the cell concentration was adjusted to 1 × 10^5^ cells/ml. The cells were fixed in 2 ml 75% ethanol (precooled at -20°C) at 4°C for 30 min. After centrifugation, the ethanol was removed, and the cells were washed twice with PBS, followed by the removal of the supernatant. Next, the cells were added with 100 µl RNase A prior to water bath at 37°C for 30 min with the avoidance of light exposure. Next, the cells were stained with 400 µl PI dye (P4170, Sigma-Aldrich Chemical Company, St. Louis, MO, USA) at 4°C for 30 min, avoiding exposure to light. Finally, the cell cycle distribution was measured through the detection of red fluorescence at an excitation wavelength of 488 nm using flow cytometry (Gallios, Beckman, Coulter, Brea, CA, USA).

Annexin-V-fluorescein isothiocyanate (FITC)/PI double staining method was utilized to detect cell apoptosis. After transfection for 48 h, the cells were washed with PBS three times and centrifuged with the removal of the supernatant. According to instructions of Annexin-V-FITC cell apoptosis detection kit (K201-100, Biovision, USA), Annexin-V-FITC/PI dye was prepared using Annexin-V-FITC, PI, and N’-a-hydroxythylpiperazine-N’-ethanesulfanic acid (HEPES) buffer at a ratio of 1:2:50. Every 100-µl dye liquor was used to resuspend 1 × 10^6^ cells, followed by oscillation and mixture. The cells were then incubated at room temperature for 15 min, added with 1 ml of HEPES buffer solution, oscillated, and mixed accordingly. FITC and PI fluorescence was detected by activating the band pass at 515 and 620 nm by a wavelength of 488 nm using flow cytometry to analyze cell apoptosis.

### Tumor Xenografts in Nude Mice

A total of 80 nude mice were divided into 16 groups (five mice in each group): five groups for intervention of LINC00883 including control, oe-NC, LINC00883, sh-NC, and sh-LINC00883 groups; five groups for intervention of miR-136 including control, NC mimic, miR-136 mimic, NC inhibitor, and miR-136 inhibitor groups; and six groups with SWOZ1 xenografts including SWOZ1, SWOZ1 + BCNU (After 10 days of SWOZ1 transplantation, mice were injected intraperitoneally with 20 mg/kg BCNU every 4 days for four times), SWOZ1 + sh-NC, SWOZ1 + sh-LINC00883, SWOZ1 + sh-NC + BCNU (After 10 days of transplantation of sh-NC-transfected SWOZ1, mice were injected intraperitoneally with 20 mg/kg BCNU every 4 days for four times), and SWOZ1 + sh-LINC00883 + BCNU (After 10 days of transplantation of sh-LINC00883-transfected SWOZ1, mice were injected intraperitoneally with 20 mg/kg BCNU every 4 days for four times) groups. Following the administration of ether for anesthetic purposes, the nude mice were subcutaneously inoculated with non-small cell glioma cell line U251 or the BCNU primary resistant cell line SWOZ1 (1 × 10^6^ cells every 200 μl) on the back of the right hind leg and fed in the same environment. The length and width of tumor were recorded in detail once every 4 days. The tumor volume = length × width²/2. On 20th day, the nude mice were subsequently euthanized in order to obtain the tumors, and three tumor samples were obtained from each group.

### Establishment of Drug-Resistant Cell Lines

Human brain glioma cell lines SWOZ1 (a primary drug-resistant cell line), SWOZ2 (a drug-sensitive cell line), and SWOZ2-carmustine (SWOZ2-BCNU, a drug-resistant cell line) were established by our laboratory and cryopreserved. BCNU (carmustine, a DNA alkylating agent) was purchased from Tianjin Pharmaceuticals (Tianjin, China). SWOZ1, SWOZ2, and SWOZ2-BCNU were cultured in RPMI 1640 culture medium containing 10% FBS, 100 U/ml penicillin, and 100 mg/L streptomycin, and incubation was carried out at 37°C with 5% CO_2_. Afterward, the expression of LINC00883, miR-136, and MRP was detected in the logarithmic and well-growing cells, while viability of each line was detected using MTT assay. The concentration of BCNU used in this assay was 20 μg/ml (19).

### Statistical Analysis

All data were processed by SPSS 21.0 statistical software (IBM Corp., Armonk, NY, USA). Measurement data were presented as mean ± standard deviation, while independent sample *t*-test was used for data analysis between two groups. One-way analysis of variance (ANOVA) was used for comparisons among multiple groups. Enumeration data were expressed by percentage and analyzed by chi square test. *p* < 0.05 was indicative of statistical significance.

## Results

### LINC00883 Competitively Binds to miR-136 to Upregulate NEK1

**Figure 1:**
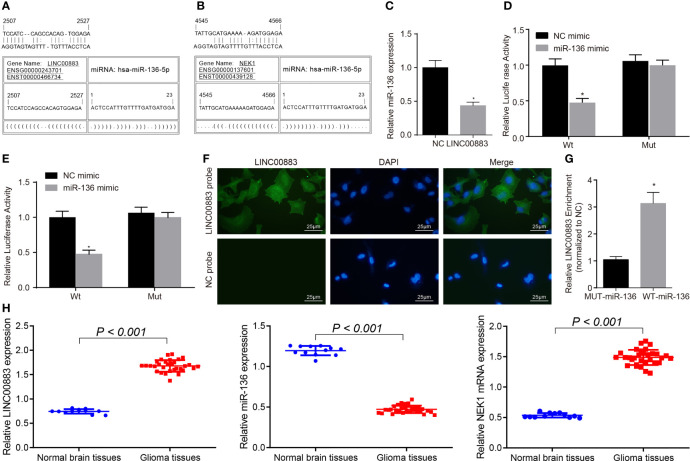
LINC00883 may increase the expression of NIMA-related kinase 1 (NEK1) by binding to miR-136. **(A)** Binding sites between LINC00883 and miR-136 as predicted by the RNA22 website. **(B)** Binding sites between NEK1 and miR-136 as predicted by the RNA22 website. **(C)** Determination of miR-136 expression after LINC00883 overexpression by RT-qPCR; *p < 0.05 *vs*. the NC group. **(D)** Binding between LINC00883 and miR-136 as confirmed by dual-luciferase reporter assay; *p < 0.05 *vs*. the NC mimic group. **(E)** miR-136 binding to NEK1 as confirmed by dual-luciferase reporter assay; *p < 0.05 *vs*. the NC mimic group. **(F)** Distribution of LINC00883 in cells as detected by RNA-fluorescence *in situ* hybridization (FISH) test (×400). **(G)** The interaction between miR-136 and LINC00883 as verified by RNA pull-down and RNA-binding protein immunoprecipitation (RIP) assays; *p < 0.05 *vs*. the MUT-miR-136 group. **(H)** The expression of LINC00883, miR-136, and NEK1 in clinical tissues as examined by RT-qPCR; *p < 0.05 *vs*. the non-neoplastic tissues. The measurement data were expressed as mean ± standard deviation and analyzed by unpaired *t*-test. Biological and technical experimental replicates were performed in triplicate.

The intersection results of the DEGs in gliomas in the GSE15824 and GSE4290 datasets and TCGA database through the jvenn website showed that only LINC00883 expression was significantly higher in glioma samples than that in the normal samples (**Supplementary Figure S1A**). A heat map of the top 10 DEGs in the GSE15824 dataset is shown in **Supplementary Figure S1B**. **Supplementary Figures S1C, D** demonstrate the expression of LINC00883 in glioma and normal samples in the GSE4290 dataset and TCGA database. The starBase website (http://starbase.sysu.edu.cn/index.php) predicted that LINC00883 and NEK1 were positively correlated (**Supplementary Figure S1E**), and that NEK1 expression was also significantly higher in glioma samples than that in the normal samples (**Supplementary Figure S1F**). The RNA22 website further predicted the presence of binding sites between LINC00883 and miR-136 (**Figure 1A**) as well as between NEK1 and miR-136 (**Figure 1B**). The above results indicated that LINC00883 may regulate NEK1 to participate in the occurrence and development of glioma through miR-136.

We then aimed to further confirm the interaction among LINC00883, miR-136, and NEK1. RT-qPCR results showed that LINC00883 overexpression reduced miR-136 expression (**Figure 1C**). Dual-luciferase reporter assay results further revealed that compared with the NC mimic group, the luciferase activity of Wt-NEK1/LINC00883 was weakened in the miR-136 mimic group (*p* < 0.05), while there was no significant difference observed in relation to the luciferase activity of Mut-NEK1/LINC00883 (*p* > 0.05) (**Figures 1D, E**), indicating that LINC00883 can specifically bind to miR-136 and that miR-136 can specifically bind to NEK1. Meanwhile, the RNA-FISH results revealed that LINC00883 was predominantly distributed in the cytoplasm (**Figure 1F**). Furthermore, the results from the RNA pull-down and RNA-binding protein immunoprecipitation (RIP) assays indicated that in comparison with the MUT-miR-136 group, there was a significant increase in LINC00883 enrichment in the WT-miR-136 group (*p* < 0.05) (**Figure 1G**), further confirming the interaction between LINC00883 and miR-136. Furthermore, the results of RT-qPCR suggested higher expression of LINC00883 and NEK1 yet poorer miR-136 expression in glioma tissues than in normal brain tissues (**Figure 1H**). The aforementioned results indicated that LINC00883 may upregulate NEK1 by competitively binding to miR-136.

### Silencing of NEK1 Reduces the Resistance of Glioma Cells to Temozolomide and Their Proliferation

**Figure 2:**
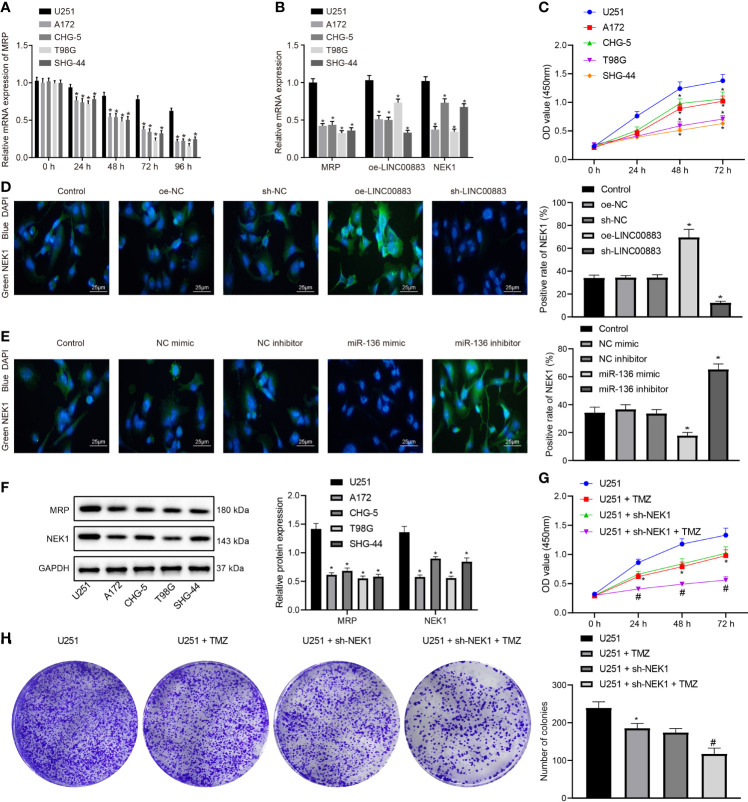
Silencing of NIMA-related kinase 1 (NEK1) blunts the resistance of U251 cells to temozolomide (TMZ) and their proliferation. **(A)** The expression of multidrug resistance-associated protein (MRP) in five glioma cell lines after treatment with TMZ (50 µM) at 0, 24, 48, 72, and 96 h as examined by RT-qPCR; *p < 0.05 *vs* . the U251 cell line. **(B)** The expression of MRP, LINC00883, and NEK1 in each cell line after treatment with TMZ (50 µM) at 72 h as detected by RT-qPCR; *p < 0.05 *vs*. the U251 cell line. **(C)** The viability of five glioma cell lines after treatment with TMZ (50 µM) at 0, 24, 48, 72, and 96 h as examined by 3-(4,5-dimethylthiazol-2-yl)-2,5-diphenyltetrazolium bromide (MTT) assay; *p < 0.05 *vs*. the U251 cell line. **(D)** The NEK1 expression in U251 cell line after ectopic expression and depletion of LINC00883 as detected by immunofluorescence (×400); *p < 0.05 *vs*. the oe-NC/sh-NC group. **(E)** The NEK1 expression in U251 cell line after upregulation and depletion of miR-136 as detected by immunofluorescence (×400); *p < 0.05 *vs*. the oe-NC/sh-NC group. **(F)** The expression of MRP and NEK1 proteins in glioma cell lines following treatment with TMZ (50 µM) for 72 h as examined by Western blot analysis. *p < 0.05 *vs*. the U251 cell line. **(G)** The viability of U251 cells upon NEK1 knockdown after treatment with TMZ (50 µM) at 0, 24, 48, 72, and 96 h as examined by MTT; *p < 0.05 *vs*. the U251 cell line; *p < 0.05 *vs*. the U251 group; # *p* < 0.05 *vs*. the U251 + sh-NEK1 group. **(H)** Colony formation of U251 cells upon NEK1 knockdown after treatment with TMZ (50 µM) for 72 h as examined by colony formation assay; *p < 0.05 *vs*. the U251 group; ^#^ *p* < 0.05 *vs*. the U251 + sh-NEK1 group. The results were measurement data, which were expressed as mean ± standard deviation and analyzed by one-way analysis of variance. Biological and technical experimental replicates were performed in triplicate.

It is well known that the resistance of glioma to TMZ is the main factor resulting in chemotherapy failure (20). For exploring the effect of LINC00883, miR-136, and NEK1 on drug resistance in glioma, we conducted a series of cell experiments. U251, A-172, CHG-5, T98G, and SHG-44 cell lines were screened by treatment with TMZ (50 µM) for 0, 24, 48, 72, and 96 h. The results depicted that the expression of resistance-related gene MRP was gradually decreased over time (**Figure 2A**), and the U251 cell line presented with higher expression of MRP, LINC00883, and NEK1 compared with the A-172, CHG-5, T98G, and SHG-44 cell lines after TMZ treatment for 72 h (*p* < 0.05) (**Figure 2B**). Additionally, MTT data manifested the strongest viability of U251 cells treated with TMZ (50 µM) for 72 h (**Figure 2C**). Therefore, the glioma cell line U251 was selected for subsequent experiments. Furthermore, the interaction among LINC00883, miR-136, and NEK1 was to be verified. NEK1 expression in U251 cells after ectopic expression and depletion of LINC00883 was detected by immunofluorescence. The results (**Figure 2D**) revealed that in contrast to the oe-NC group, the oe-LINC00883 group had increased NEK1 expression, while the sh-LINC00883 group showed decreased NEK1 expression compared with that in the sh-NC group. Meanwhile, immunofluorescence was used to examine NEK1 expression in U251 cells after ectopic expression and depletion of miR-136. The results revealed that the miR-136 mimic group presented with significantly lower NEK1 expression than the NC mimic group, while the miR-136 inhibitor group exhibited evidently higher levels in NEK1 expression than the NC inhibitor group (**Figure 2E**). In addition, the results of Western blot analysis documented that MRP and NEK1 protein expression was obviously higher in the U251 cell line than that in other cell lines following treatment with TMZ (50 µM) for 72 h (**Figure 2F**). Furthermore, MTT and colony formation assays indicated that U251 cells lost drug resistance upon NEK1 knockdown after treatment with TMZ (50 µM) for 72 h, with diminished proliferation (**Figures 2G, H**). Therefore, U251 cells may have the highest expression of LINC00883 and NEK1 and the strongest drug resistance after TMZ treatment. Silencing of NEK1 can attenuate the resistance of U251 cells to TMZ and their proliferation.

### LINC00883 Silencing or miR-136 Overexpression Suppresses Glioma Cell Proliferation While Improving Cell Apoptosis

**Figure 3:**
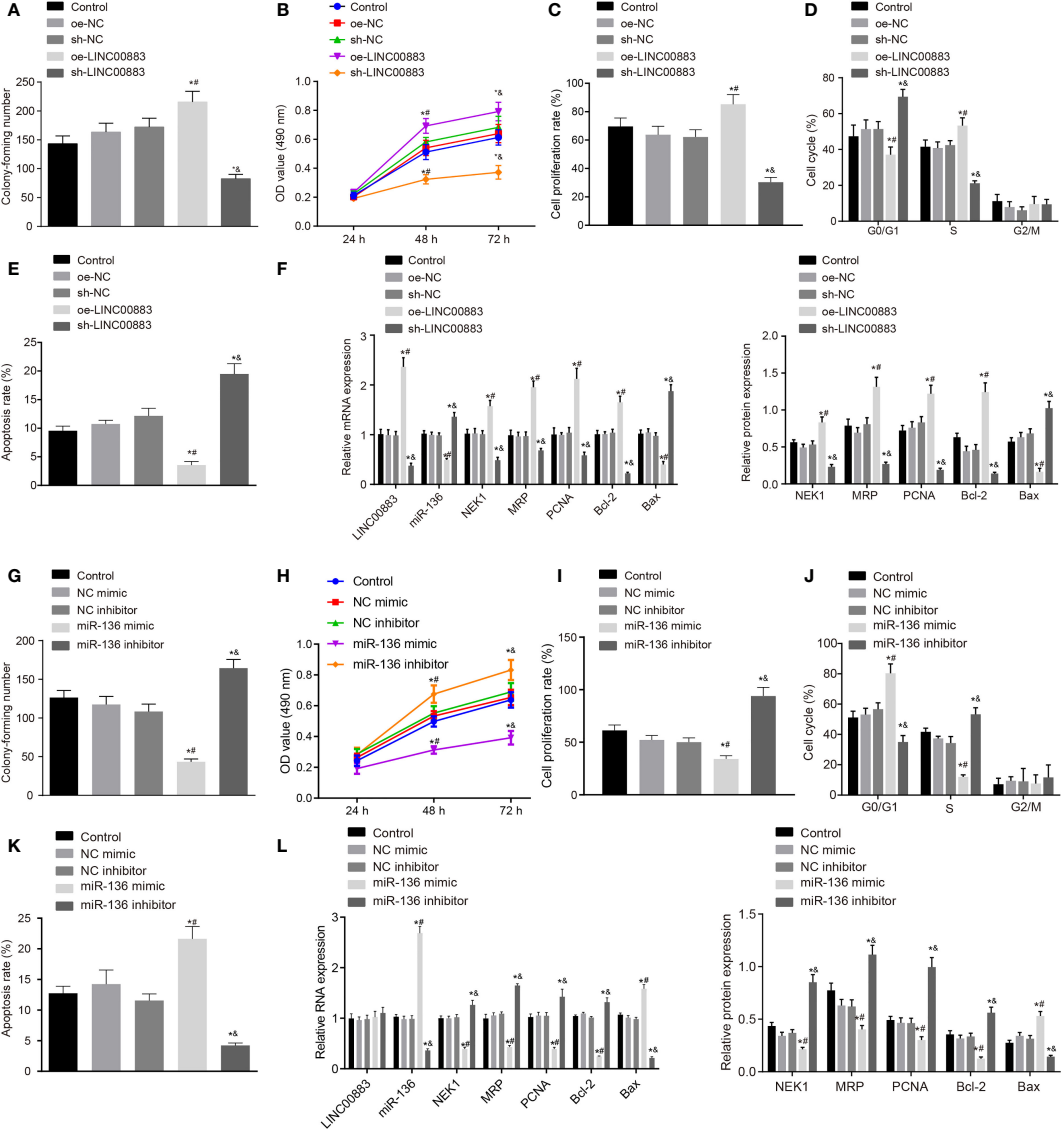
Silencing of LINC00883 or overexpressed miR-136 inhibits colony formation and cell proliferation but enhances cell apoptosis in U251 cells. **(A)** The effect of ectopic expression and depletion of LINC00883 on colony formation of cells as examined by colony formation assay. **(B)** Viability of U251 cells when LINC00883 was upregulated or silenced as examined by 3-(4,5-dimethylthiazol-2-yl)-2,5-diphenyltetrazolium bromide (MTT) assay. **(C)** The effect of ectopic expression and depletion of LINC00883 on U251 cell proliferation as detected by 5-ethynyl-2′-deoxyuridine (EdU) assay. **(D)** The effect of ectopic expression and depletion of LINC00883 on U251 cell cycle distribution as detected by flow cytometry. **(E)** The effect of ectopic expression and depletion of LINC00883 on U251 cell apoptosis as detected by flow cytometry. **(F)** The expression of LINC00883, miR-136, Bax, NIMA-related kinase 1 (NEK1), proliferating cell nuclear antigen (PCNA), multidrug resistance-associated protein (MRP), and Bcl-2 in U251 cells in response to ectopic expression and depletion of LINC00883 as examined by RT-qPCR and Western blot analysis. **(G)** The impact of ectopic expression and depletion of miR-136 on colony formation of cells by colony formation assay. **(H)** Viability of U251 cells after restoration or disruption of miR-136 as examined by MTT assay. **(I)** Cell proliferation mediated by ectopic expression or depletion of miR-136 in each group as assessed by EdU assay. **(J)** Cell cycle distribution in U251 cells after restoration or disruption of miR-136 as assessed by flow cytometry. **(K)** U251 cell apoptosis in response to ectopic expression or inhibition of miR-136 as assessed by flow cytometry. **(L)** The expression of LINC00883, miR-136, Bax, NEK1, PCNA, MRP, and Bcl-2 in cells in response to ectopic expression or depletion of miR-136 as measured by RT-qPCR and Western blot analysis. *p < 0.05 *vs*. the control group; ^#^ *p* < 0.05 *vs*. the oe-NC or NC mimic group; ^&^ *p* < 0.05 *vs*. the sh-NC or NC inhibitor group. The above data were expressed as mean ± standard deviation and analyzed by one-way analysis of variance. Biological and technical experimental replicates were performed in triplicate.

To explore the effects of LINC00883 on the cellular behavior of glioma, LINC00883 was overexpressed or silenced in U251 cells. The oe-LINC00883 group presented with increased cell proliferation (**Figures 3A–C** and **Supplementary Figures S2A, B**), lower number of cells in G0/G1 phase and more cells in S phase (**Figure 3D**), and decreased cell apoptosis (**Figure 3E**) than those in the oe-NC group, which was opposite in the sh-LINC00883 group compared to the sh-NC group (all *p* < 0.05). Lastly, RT-qPCR and Western blot analysis (**Figure 3F** and **Supplementary Figure S2C**) displayed that compared with those in the oe-NC group, there was a significant downregulation in the expression of Bax, while those of NEK1, PCNA, MRP, and Bcl-2 were upregulated in the oe-LINC00883 group (*p* < 0.05), but the sh-LINC00883 group presented the opposite trends in comparison with the sh-NC group (*p* < 0.05).

Next, for proliferation, U251 cells were treated with miR-136 mimic or inhibitor. The miR-136 mimic group exhibited decreased cell proliferation (**Figures 3G–I** and **Supplementary Figures S2D, E**), more cells in G0/G1 phase while less cells in S phase (**Figure 3J**), and increased cell apoptosis (**Figure 3K**) than those in the NC mimic group, while the miR-136 inhibitor group showed opposite changes in comparison with the NC inhibitor group (all *p* < 0.05). Finally, RT-qPCR and Western blot analysis (**Figure 3L** and **Supplementary Figure S2F**) revealed that in contrast to the NC mimic group, there was a significant increase in the expression of Bax, while that of NEK1, PCNA, MRP, and Bcl-2 was decreased in the miR-136 group (all *p* < 0.05), which was reverse in the miR-136 inhibitor group compared with the NC inhibitor group (all *p* < 0.05).

The aforementioned results suggested that the silencing of LINC00883 or overexpression of miR-136 inhibited glioma cell proliferation while promoting cell apoptosis.

### Downregulation of LINC00883 or Upregulation of miR-136 Suppresses the Tumorigenicity of U251 Cells

**Figure 4:**
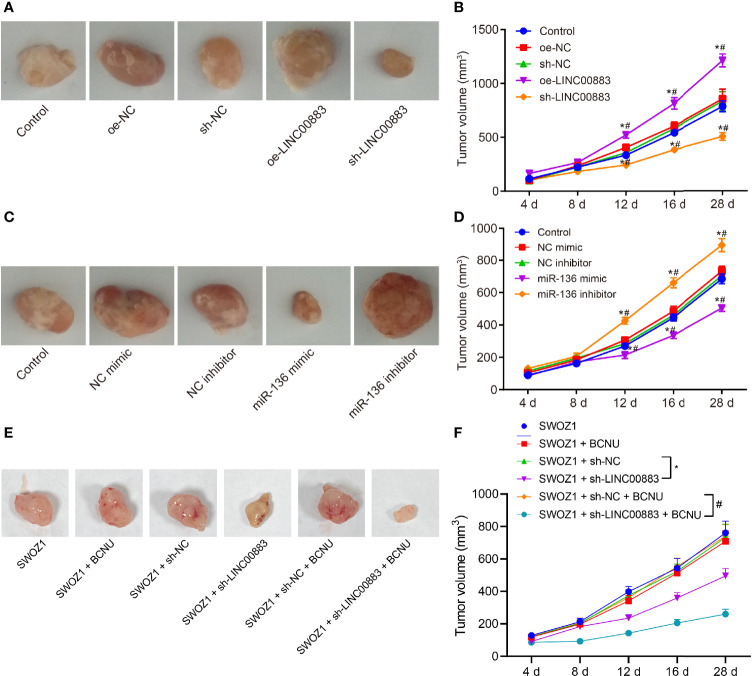
Silencing of LINC00883 or overexpression of miR-136 represses the tumorigenicity of drug-resistant cell line U251. **(A)** The transplanted tumors of nude mice after overexpressing or silencing LINC00883. **(B)** The effect of overexpressing or silencing LINC00883 on tumor volume. **(C)** The transplanted tumors of nude mice after overexpressing or silencing miR-136. **(D)** The effect of overexpressing or silencing miR-136 on tumor volume. **(E)** Representative images showing xenografts in nude mice inoculated with SWOZ1 cells with sh-LINC00883 or/and treated with BCNU. **(F)** Tumor volume of mice inoculated with SWOZ1 cells with sh-LINC00883 or/and treated with BCNU. *p < 0.05 *vs*. the control or SWOZ1 + sh-NC group; ^#^ *p* < 0.05 *vs*. the oe-NC, SWOZ1 + sh-NC + BCNU or NC mimic group; *vs*. the sh-NC or NC inhibitor group. The values of tumor volume were measurement data, which were expressed as mean ± standard deviation and analyzed by repeated-measures analysis of variance. n = 5 mice in each group. Biological and technical experimental replicates were performed in triplicate.

Tumor xenograft in nude mice was applied to analyze the effects of LINC00883 on tumor growth through the injection of U251 cell line overexpressing or silencing LINC00883. Based on the results, compared with the oe-NC group, the tumor growth rate in the oe-LINC00883 group was elevated, while that in the sh-LINC00883 group was reduced in contrast to that in the sh-NC group (all *p* < 0.05) (**Figures 4A, B**).

Subsequently, U251 cells with miR-136 mimic or inhibitor were injected into nude mice to investigate the effects of miR-136 on tumor growth. The results (**Figures 4C, D**) showed that in contrast to the NC mimic group, the miR-136 mimic group presented with lower tumor growth rate, while the miR-136 inhibitor group exhibited higher tumor growth rate than the NC inhibitor group (all *p* < 0.05).

In addition, the BCNU primary drug-resistant cell line SWOZ1 with LINC00883 silencing was used for subcutaneous transplantation tumor experiments, followed by BCNU treatment. As shown in **Figures 4E, F**, relative to the SWOZ1 + sh-NC group, tumor growth was decreased in the SWOZ1 + sh-LINC00883 group. In comparison to that in the SWOZ1 + sh-NC + BCNU group, tumor growth was suppressed in the SWOZ1 + sh-LINC00883 + BCNU group. Additionally, tumor growth was slower in the SWOZ1 + sh-LINC00883 + BCNU group than that in the SWOZ1 + sh-LINC00883 group. These results indicate that tumorigenicity of human glioma U251 cell line could be inhibited through the downregulation of LINC00883 or upregulation of miR-136 and that silencing of LINC00883 can increase the sensitivity of drug-resistant cell lines to BCNU.

### LINC00883 Impairs miR-136-Dependent NEK1 Inhibition to Promote the Drug Resistance of Glioma Cells

**Figure 5:**
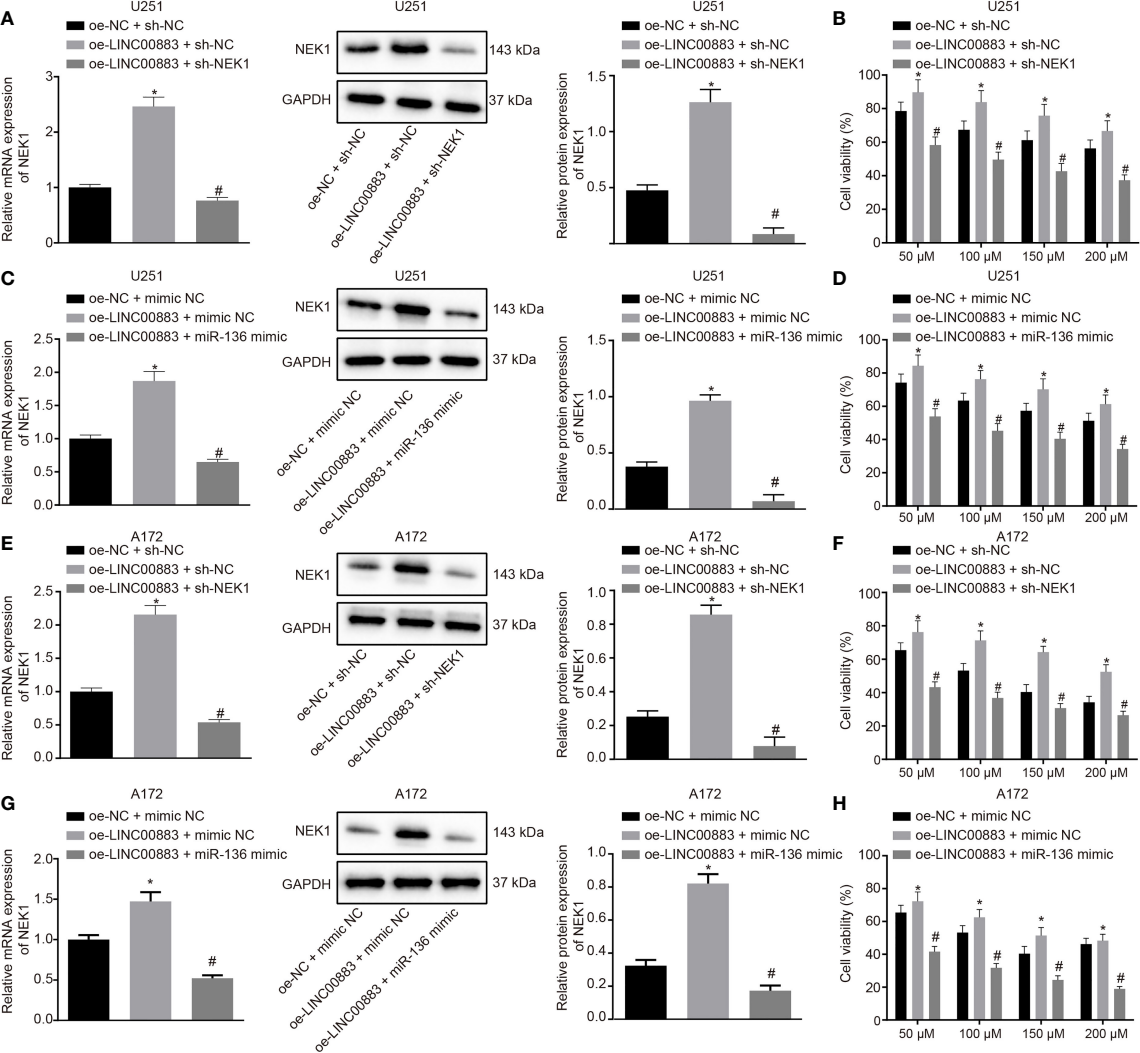
LINC00883 blocks miR-136-dependent NIMA-related kinase 1 (NEK1) inhibition, thereby inducing drug resistance of glioma cells. **(A)** The NEK1 expression in U251 cell line cotransfected with oe-LINC00883 and sh-NEK1/sh-NC as examined by RT-qPCR and Western blot analysis. **(B)** The viability of U251 cell line cotransfected with oe-LINC00883 and sh-NEK1/sh-NC as examined by 3-(4,5-dimethylthiazol-2-yl)-2,5-diphenyltetrazolium bromide (MTT) assay. **(C)** The NEK1 expression in U251 cell line cotransfected with oe-LINC00883 and miR-136 mimic/NC mimic as measured by RT-qPCR and Western blot analysis. **(D)** The viability of U251 cell line cotransfected with oe-LINC00883 and miR-136 mimic/NC mimic as examined by MTT assay. **(E)** The NEK1 expression in A-172 cell line cotransfected with oe-LINC00883 and sh-NEK1/sh-NC as examined by RT-qPCR and Western blot analysis. **(F)** The viability of A-172 cell line cotransfected with oe-LINC00883 and sh-NEK1/sh-NC as examined by MTT assay. **(G)** The NEK1 expression in A-172 cell line cotransfected with oe-LINC00883 and miR-136 mimic/NC mimic by RT-qPCR and Western blot analysis. **(H)** Viability of A-172 cell line cotransfected with oe-LINC00883 and miR-136 mimic/NC mimic as examined by MTT assay. *p < 0.05 *vs*. the oe-NC + sh-NC group; ^#^ *p* < 0.05 *vs*. the oe-LINC00883 + sh-NC. The above data were expressed as mean ± standard deviation and analyzed by one-way analysis of variance. Biological and technical experimental replicates were performed in triplicate.

The above results indicated that LINC00883 and miR-136 regulated the proliferation, cell cycle distribution, apoptosis, and tumorigenicity of glioma U251 cell line. Next, we explored the regulatory role of LINC00883/miR-136/NEK1 axis in drug resistance of U251 cells. U251 cells were cotransfected with oe-NC + sh-NC, oe-LINC00883 + sh-NC, and oe-LINC00883 + sh-NEK1 for 24 h. Next, RT-qPCR and Western blot analysis (**Figure 5A**) exhibited that the oe-LINC00883 + sh-NC group presented with higher mRNA and protein expression of NEK1 than the oe-NC + sh-NC group, while the oe-LINC00883 + sh-NEK1 group exhibited lower mRNA and protein expression of NEK1 than the oe-LINC00883 + sh-NC group (all *p* < 0.05). Subsequently, U251 cells were treated with TMZ with different concentrations of 50, 100, 150, and 200 µM, respectively, for 72 h. MTT assay (**Figure 5B**) revealed that following treatment with TMZ, the oe-LINC00883 + sh-NC group presented with significantly increased cell viability in comparison to that in the oe-NC + sh-NC group, while the oe-LINC00883 + sh-NEK1 group showed decreased cell viability than that in the oe-LINC00883 + sh-NC group (all *p* < 0.05). Those data suggested that LINC00883 enhances the drug resistance of U251 cells by upregulating NEK1.

In addition, U251 cells were cotransfected with oe-NC + mimic N, oe-LINC00883 + mimic NC, and oe-LINC00883 + miR-136 mimic for 24 h. RT-qPCR and Western blot analysis were employed to examine NEK1 mRNA and protein expression in cells, with the results shown in **Figure 5C**. In contrast to the oe-NC + mimic NC group, the oe-LINC00883 + mimic NC group presented with significantly increased NEK1 mRNA and protein expression. The oe-LINC00883 + miR-136 mimic group showed evidently reduced NEK1 mRNA and protein expression compared with the oe-LINC00883 + mimic NC group (all *p* < 0.05), suggesting that LINC00883 upregulated NEK1 by inhibiting miR-136. Subsequently, U251 cells were treated with TMZ with concentrations of 50, 100, 150, and 200 μM, respectively, for 72 h, followed by MTT assay to examine cell viability. The results (**Figure 5D**) revealed that following TMZ treatment, cell viability was higher in the oe-LINC00883 + mimic NC group than that in the oe-NC + mimic NC group, while the oe-LINC00883 + miR-136 mimic group had lower cell viability than that in the oe-LINC00883 + mimic NC group (all *p* < 0.05). Those data suggested that LINC00883 enhances the drug resistance of U251 cells by inhibiting miR-136.

Moreover, another glioma cell line A-172 was selected to conduct the above experiments, and the obtained results were consistent with those from the U251 cell line (**Figures 5E–H**). The aforementioned results suggest that LINC00883 could enhance the drug resistance of glioma cells by blocking miR-136-dependent NEK1 inhibition.

### LINC00883 Silencing Suppresses the Viability of Drug-Resistant Glioma Cells

The primary drug-resistant glioma cell line SWOZ1, the drug-sensitive glioma cell line SWOZ2, as well as the drug-resistant glioma cell line SWOZ2-BCNU were selected for LINC00883 loss-of-function experiments in order to substantiate the role of LINC00883 in the drug resistance of glioma cells.

**Figure 6:**
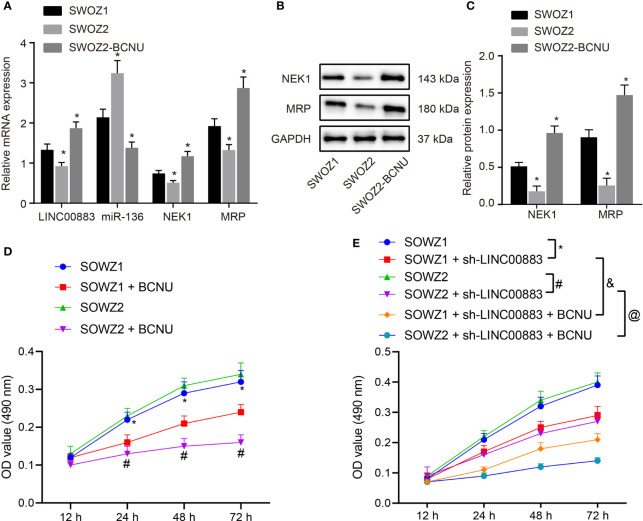
LINC00883 silencing inhibits the viability of drug-resistant glioma cells. **(A)** The expression of LINC00883, miR-136, NIMA-related kinase 1 (NEK1) mRNA, and multidrug resistance-associated protein (MRP) mRNA in cell lines SWOZ1, SWOZ2, and SWOZ2-BCNU as detected by RT-qPCR. **(B)** The protein bands of NEK1, MRP, and GAPDH in cell lines SWOZ1, SWOZ2, and SWOZ2-BCNU as examined by Western blot analysis. **(C)** The protein levels of NEK1 and MRP in cell lines SWOZ1, SWOZ2, and SWOZ2-BCNU as examined by Western blot analysis. **(D)** The viability of SWOZ1, SWOZ2, and SWOZ2-BCNU cells after BCNU treatment as examined by 3-(4,5-dimethylthiazol-2-yl)-2,5-diphenyltetrazolium bromide (MTT) assay. **(E)** The viability of cell lines SWOZ1, SWOZ2, and SWOZ2-BCNU treated with BCNU after LINC00883 silencing as examined by MTT assay. *p < 0.05 *vs*. the SWOZ1 cell line; ^#^ *p* < 0.05 *vs*. the SOWZ2 cell line; ^&^ *p* < 0.05 *vs*. the SOWZ1 + sh-LINC00883 group; ^@^ *p* < 0.05 *vs*. the SOWZ2 + sh-LINC00883 group. The results of RT-qPCR, Western blot analysis, and MTT assay were measurement data, which were expressed as mean ± standard deviation. The results of RT-qPCR and Western blot analysis were analyzed by one-way analysis of variance, and the results of MTT assay were analyzed by repeated-measures analysis of variance. Biological and technical experimental replicates were performed in triplicate.

RT-qPCR (**Figure 6A**) revealed that compared with SWOZ1 cell line, the expression of miR-136 was decreased in SWOZ2-BCNU cell line where LINC00883, NEK1, and MRP expression was increased significantly (all *p* < 0.05). There was a notable elevation in the expression of miR-136 in SWOZ2 cell line, while those of LINC00883, NEK1, and MRP were significantly decreased as compared to SWOZ1 cell line (all *p* < 0.05). The results from Western blot analysis revealed that compared with SWOZ1 cell line, NEK1 and MRP protein levels were significantly increased in SWOZ2-BCNU cell line but decreased in SWOZ2 cell line (**Figures 6B, C**) (all *p* < 0.05). The viability of those cell lines treated with BCNU was evaluated by MTT assay (**Figure 6D**), which revealed that after BCNU treatment, the viability of SWOZ2-BCNU cell line was significantly decreased (*p* < 0.05). Subsequently, MTT assay was conducted to examine the effect of the silencing of LINC00883 on the growth of these cell lines. The results showed that silencing of LINC00883 reduced the proliferation of the two cell lines SWOZ1 and SWOZ2 and induced a significant decrease in cell viability and an enhancement of sensitivity to BCNU in SWOZ1 cell line (**Figure 6E**) (*p* < 0.05). These results suggested that LINC00883 silencing enhanced the sensitivity of drug-resistant glioma cells to BCNU.

## Discussion

Glioma is a primary human disorder characterized by invasive growth and aggressive proliferation. The treatment protocol for patients with glioma includes a series of complicated procedures, with patients undergoing numerous surgical procedures, only to be met with a prognosis of no more than 24 months (21, 22). miRNAs and lncRNAs have been found to have implications in the progression of several human disorders; however, there is still a lack of distinct delineation between them (23). Our study was designed and conducted with a conclusion premised upon the notion that silencing LINC00883 could enhance the negative regulation of NEK1 by miR-136 and effectively reduce the malignant tumor characteristics of glioma, while reducing the risk of drug resistance.

A key finding from the present study found that LINC00883 could bind to miR-136, and miR-136 could negatively regulate its target gene, NEK1. Such a regulatory model regarding ceRNA–miRNA–mRNA interaction has previously been documented whereby reports have indicated that LINC00883 functions as a “sponge” for particular miRNAs, by which the targets of those miRNAs can be expressed (10). In addition, it has been demonstrated that the lncRNAs share a relationship with miRNAs that compete with its target gene, while indicating that its target gene expression could be further regulated in an indirect manner, possibly providing a novel perspective in elucidating tumor pathogenesis (24). Similarly, lncRNA regulator of reprogramming (ROR) acts as a ceRNA and binds to miR-145 to modulate Nanog expression, resulting in the suppression of tumorigenicity in pancreatic cancer (25). Hong et al. (26) have identified that through the competitive binding of miR-217, lncRNA HOX transcript antisense RNA (HOTAIR) promoted tumorigenesis in renal cell carcinoma *via* HIF-1α expression.

In this study, we found that the silencing of LINC00883 or upregulation of miR-136 could suppress the proliferation, tumorigenicity, and drug resistance as well as induce apoptosis. The experimental data suggested that regarding interference, there was a decrease in the expression levels of PCNA and Bax and increase in Bcl-2 expression. According to systematic functional screening and data analysis presented in a prior study, overexpression of miR-136 has been validated to exert an inhibitory effect on proliferation of A-172 and LN405 cell lines in glioblastoma multiforme, which is a type of malignant glioma (27). The proapoptotic effects of miR-136 on glioma cells in connection with the induction of chemotherapy have been revealed to have been associated with Bcl-2, a well-known apoptosis inhibitor, indicating the tumor-suppressive role of miR-136 in glioma (28). At present, there exists a wide consensus acknowledging Bcl-2 to be a classical apoptosis-related gene that can stabilize the mitochondrial membrane in combination with Bax, thus promoting survival and inhibiting apoptosis (29). An interesting finding exhibits a similar regulatory mechanism, as lncRNA CRNDE serves as a ceRNA that binds to miR-136-5p, by which glioma malignancy is promoted *via* miR-136-5p-mediated Bcl-2 (30).

Furthermore, there was a significant reduction in drug resistance of U251 cell through miR-136-5p-mediated silencing of NEK1 by inhibiting LINC00883, which was evidenced by the decreased MRP level. Besides, those findings were further verified in A-172 cell line. Consistently, miR-136 has been revealed to reduce TMZ resistance by targeting astrocyte-elevated gene-1 (AEG-1) in glioma (31). In addition, miR-136 has been proven to be capable of suppressing chemoresistance of glioma cells (14). BCNU, a type of DNA alkylating agent, has been widely applied in surgery for the treatment of glioma associated with enhanced survival advantages, as it can significantly prolong a patient’s median survival time by approximately 2~4 months (32). In a former study, it revealed that the drug resistance of glioma could be reduced through the downregulation of drug-resistant proteins P-gp and MRP1 by low-intensity ultrasound (LIUS) (33). The MRP family is composed of MRP1, MRP3, and MRP5, all of which are essential for the metabolic equilibrium of the brain and in providing protection from toxins (34). Similarly, miR-136 has also been found to sensitize U251 glioma cells to TMZ by regulating target AEG-1 (31).

## Conclusion

**Figure 7:**
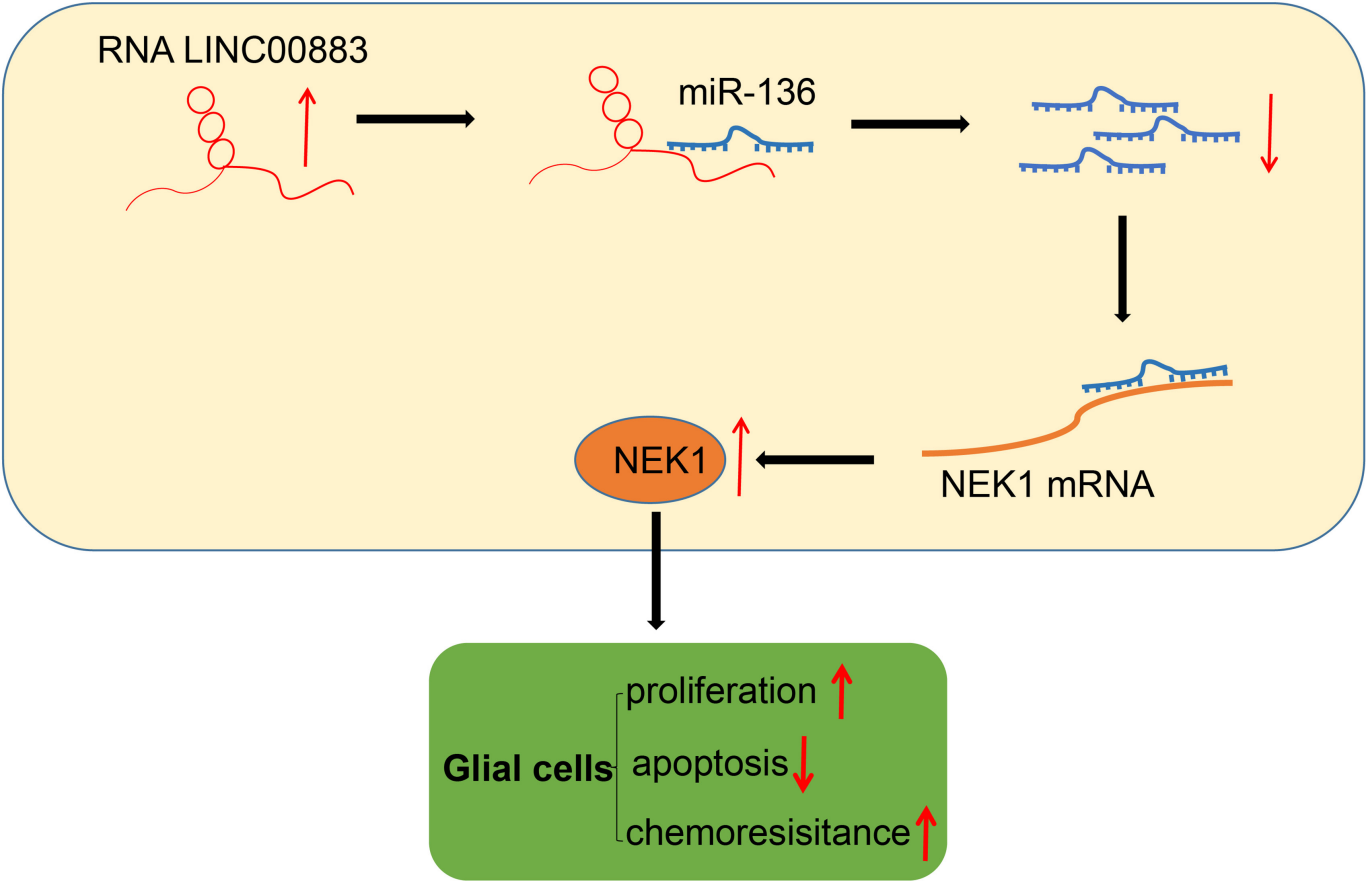
Schematic diagram of the mechanism by which LINC00883 affects the proliferation and drug resistance of glioma cells. LINC00883 competitively binds to miR-136 and attenuates the binding of miR-136 to NIMA-related kinase 1 (NEK1), thus enhancing glioma cell proliferation and drug resistance but reducing cell apoptosis.

To summarize our findings, we were able to evidently demonstrate that LINC00883 functions as a ceRNA of miR-136 to upregulate NEK1 (**Figure 7**). Our results also highlighted that LINC00883 could serve as a potential prognostic biomarker and/or therapeutic target in glioma, which was further supported *via* the elucidation that inhibition of LINC00883 resulted in the suppression of tumorigenicity and multidrug resistance while inducing the apoptosis of glioma cells. Therefore, our study provided further insight into the molecular mechanisms associated with the pathogenesis of glioma and anti-chemoresistant mechanism. Still, further studies with specimens from glioma-diagnosed patients are needed to confirm the clinical application of the LINC00883/miR-136/NEK1 axis in treating glioma. In addition, U251 is not a great glioma cell line accompanied by a lot of issues. Meanwhile, in this study, we performed *in vivo* experimental model validation in subcutaneous xenograft tumor models, and the limitations were obvious, which partly affected the application of the research conclusions. Considering these limitations, additional studies are a prerequisite to validate the current findings and expand the translational potential of this direction.

## Data Availability Statement

The datasets presented in this study can be found in online repositories. The names of the repository/repositories and accession number(s) can be found in the article/**Supplementary Material**.

## Ethics Statement

The studies involving human participants were reviewed and approved by the Ethics Committee of the Second Affiliated Hospital of Harbin Medical University. The patients/participants provided their written informed consent to participate in this study. The animal study was reviewed and approved by the Animal Ethics Committee of the Second Affiliated Hospital of Harbin Medical University.

## Author Contributions

YL and XG designed the study. YL and XG collated the data, carried out data analyses, and produced the initial draft of the article. YL and XG contributed to drafting the article. All authors contributed to the article and approved the submitted version.

## Conflict of Interest

The authors declare that the research was conducted in the absence of any commercial or financial relationships that could be construed as a potential conflict of interest.

## Publisher’s Note

All claims expressed in this article are solely those of the authors and do not necessarily represent those of their affiliated organizations, or those of the publisher, the editors and the reviewers. Any product that may be evaluated in this article, or claim that may be made by its manufacturer, is not guaranteed or endorsed by the publisher.

## Supplementary Material

Supplementary Figure 1 Bioinformatics analysis predicts the regulatory mechanism of LINC00883 in glioma. **(A)** The intersection results of the DEGs in gliomas in the GSE15824 and GSE4290 datasets and TCGA database by the jvenn website. **(B)** A heat map of the top 10 DEGs in the GSE15824 dataset. The abscissa refers to sample number, and the ordinate refers to names of DEGs. The upper dendrogram refers to cluster analysis of sample types, and the color band represents the sample type. The left dendrogram refers to the cluster analysis of DEGs. Each block represents the expression of gene in one sample, and the upper right histogram refers to color gradation. **(C)** Expression of LINC00883 in glioma and normal samples in the GSE4290 dataset. **(D)** Expression of LINC00883 in glioma and normal samples in TCGA database. **(E)** Correlation of LINC00883 and NEK1 in glioma samples analyzed by the starBase website. **(F)** Expression of NEK1 in glioma and normal samples in TCGA database.

Supplementary Figure 2 Representative images and representative western blots. **(A)** Representative images of colony formation assay after ectopic expression and depletion of LINC00883. **(B)** Representative images of EdU assay of U251 cells after ectopic expression and depletion of LINC00883 (× 200). **(C)** Representative western blots of Bax, NEK1, PCNA, MRP and Bcl-2 in U251 cells in response to ectopic expression and depletion of LINC00883. **(D)** Representative images of colony formation assay after ectopic expression and depletion of miR-136. **(E)** Representative images of EdU assay of U251 cells after ectopic expression or depletion of miR-136 (× 200). **(F)** Representative western blots of Bax, NEK1, PCNA, MRP and Bcl-2 in U251 cells in response to ectopic expression or depletion of miR-136.
