# Supplementary figures and images for: LINC00883 Promotes Drug Resistance of Glioma Through a microRNA-136/NEK1-Dependent Mechanism
Source: Front Oncol. 2022 Jan 10;11:692265. doi: 10.3389/fonc.2021.692265 (PMC8785904; doi:10.3389/fonc.2021.692265)

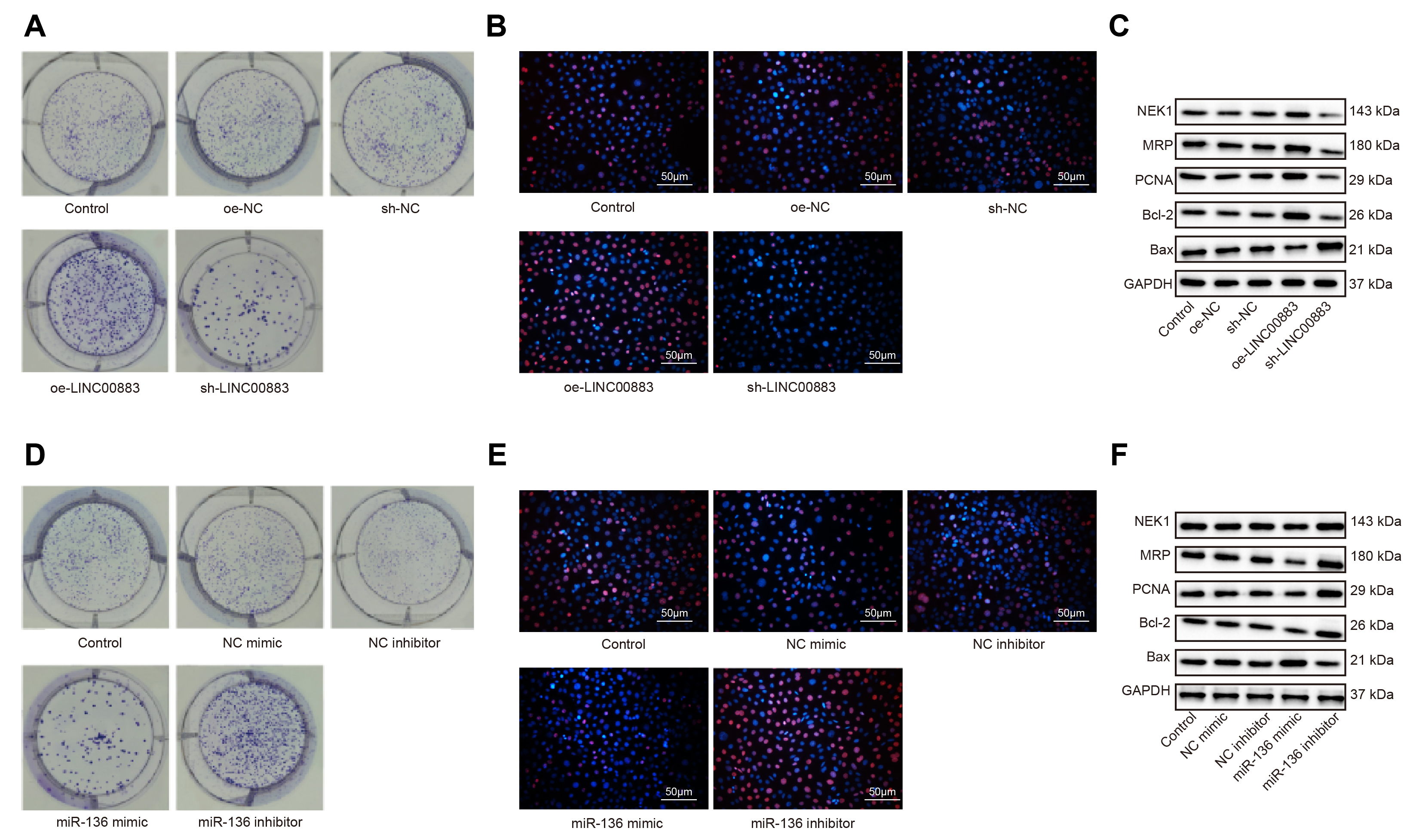

Supplement: Supplementary Figure 1 — Bioinformatics analysis predicts the regulatory mechanism of LINC00883 in glioma. (A) The intersection results of the DEGs in gliomas in the GSE15824 and GSE4290 datasets and TCGA database by the jvenn website. (B) A heat map of the top 10 DEGs in the GSE15824 dataset. The abscissa refers to sample number, and the ordinate refers to names of DEGs. The upper dendrogram refers to cluster analysis of sample types, and the color band represents the sample type. The left dendrogram refers to the cluster analysis of DEGs. Each block represents the expression of gene in one sample, and the upper right histogram refers to color gradation. (C) Expression of LINC00883 in glioma and normal samples in the GSE4290 dataset. (D) Expression of LINC00883 in glioma and normal samples in TCGA database. (E) Correlation of LINC00883 and NEK1 in glioma samples analyzed by the starBase website. (F) Expression of NEK1 in glioma and normal samples in TCGA database. [file Image_1.jpg]

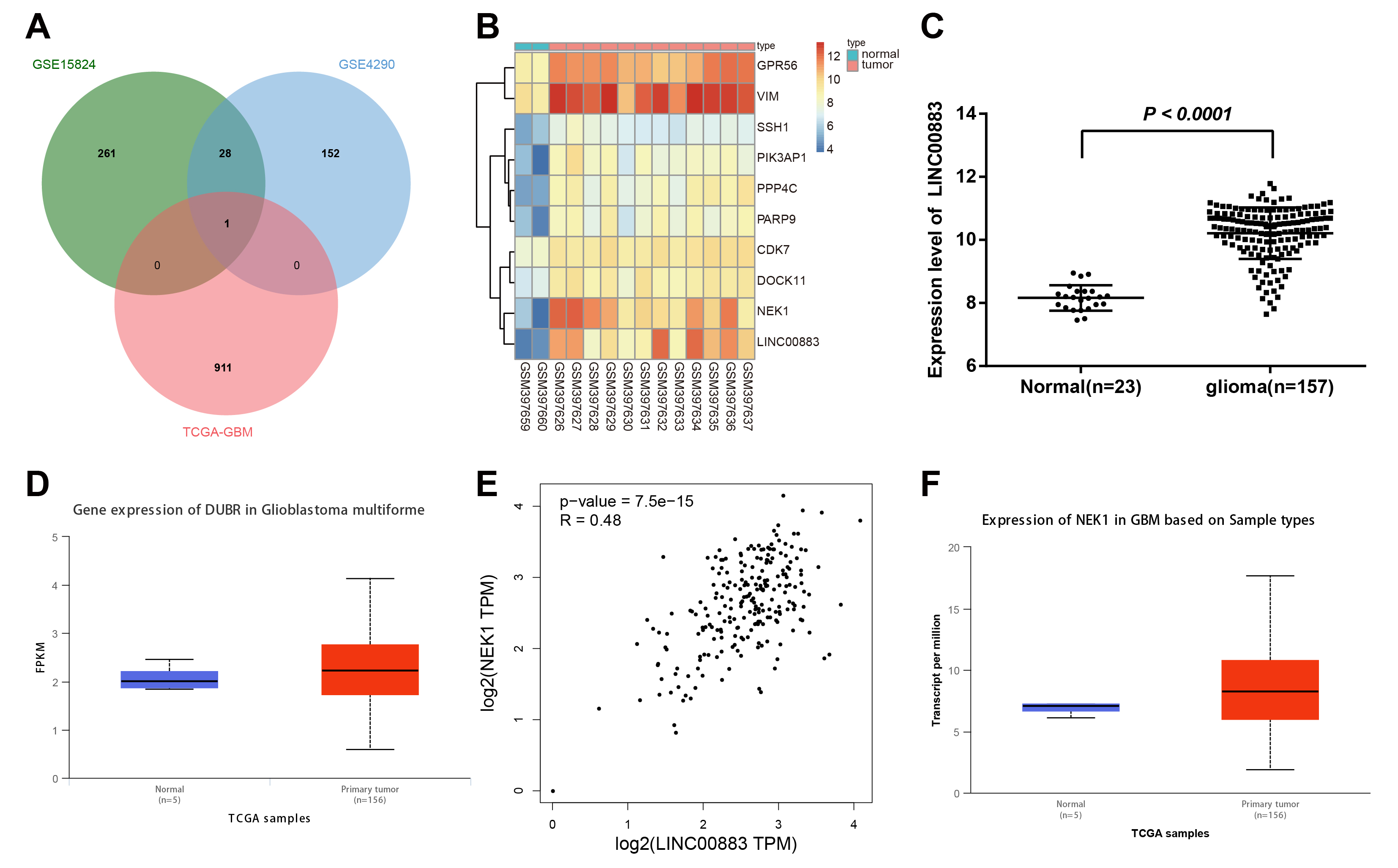

Supplement: Supplementary Figure 2 — Representative images and representative western blots. (A) Representative images of colony formation assay after ectopic expression and depletion of LINC00883. (B) Representative images of EdU assay of U251 cells after ectopic expression and depletion of LINC00883 (× 200). (C) Representative western blots of Bax, NEK1, PCNA, MRP and Bcl-2 in U251 cells in response to ectopic expression and depletion of LINC00883. (D) Representative images of colony formation assay after ectopic expression and depletion of miR-136. (E) Representative images of EdU assay of U251 cells after ectopic expression or depletion of miR-136 (× 200). (F) Representative western blots of Bax, NEK1, PCNA, MRP and Bcl-2 in U251 cells in response to ectopic expression or depletion of miR-136. [file Image_2.jpeg]
