# Supplementary material for: LINC00883 Promotes Drug Resistance of Glioma Through a microRNA-136/NEK1-Dependent Mechanism
Source: Front Oncol. 2022 Jan 10;11:692265. doi: 10.3389/fonc.2021.692265 (PMC8785904; doi:10.3389/fonc.2021.692265)
Supplement: Supplementary file 3 [file Table_1.docx]

**Supplementary Table 1** Primer sequences for RT-qPCR

| Gene | Sequence (5'-3') |
| --- | --- |
| LINC00883 | Forward: 5'-CTGCAGAGAGCATCCTCATCCAT-3' |
|  | Reverse: 5'-CACCTGGGAATGCGCTTGCTG-3' |
| miR-136 | Forward: 5'-ACTCCATTTGTTTTGATGATGGA-3' |
|  | Reverse: universal primer in the kit |
| NEK1 | Forward: 5'-AATGCTCAGAAAGGCGTTTTGT-3' |
|  | Reverse: 5'-GGGGTCCCTATGCAAGTTCG-3' |
| PCNA | Forward: 5'-AGGTGTTGGAGGCACTCAAG-3' |
|  | Reverse: 5'-AGGTATCCGCGTTATCTTCG-3' |
| MRP | Forward: 5'-GCGACGGCCCTTATGATCC-3' |
|  | Reverse: 5'-GCTTGAGGGGCTCATTCAAAATC-3' |
| Bcl-2 | Forward: 5'-GTGGAGGAGCTCTTCAGGGA-3' |
|  | Reverse: 5'-AGGCACCCAGGGTGATGCAA-3' |
| Bax | Forward: 5'-GCCCACCAGCTCTGAGCAGATCAT-3' |
|  | Reverse: 5'-CGGCAAT-CATCCTCTGCAGC-3' |
| U6 | Forward: 5'-CTCGCTTCGGCAGCACATA-3' |
|  | Reverse: universal primer in the kit |
| GAPDH | Forward: 5'-GACTCATGACCACAGTCCATGC-3' |
|  | Reverse: 5'-AGAGGCAGGGATGATGTTCTG-3' |

Note: RT-qPCR, reverse transcription quantitative polymerase chain reaction; miR-136, microRNA-136; NEK1, NIMA-related kinase 1; PCNA, proliferating cell nuclear antigen; MRP, multidrug-resistance associated protein; Bcl-2, B-cell lymphoma-2; Bax, Bcl-2 associated protein X; GAPDH, glyceraldehyde-3-phosphate dehydrogenase.

**Supplementary Table 2** Identification of IncRNA

| LncRNA | Ensemble ID | Length |
| --- | --- | --- |
| LINC00883 | ENST00000466734 | 2737 |

Note: lncRNA, long noncoding RNA.
